# Interpretation of sonotubometric data based on phase-shift detection

**DOI:** 10.1186/s40463-016-0151-5

**Published:** 2016-06-10

**Authors:** Yaw Amoako-Tuffour, Philip Garland, Manohar Bance

**Affiliations:** Faculty of Medicine, Dalhousie University, 1459 Oxford Street, Halifax, Nova Scotia, B3H 4R2 Canada; Department of Mechanical Engineering, University of New Brunswick, 15 Dineen Drive, P.O. Box 4400, Fredericton, New Brunswick E3B 5A3 Canada; Division of Otolaryngology – Head and Neck Surgery, Dalhousie University, 3rd Floor Dickson Building, VG Site, QE II Health Sciences Centre, 5820 University Ave, Halifax, Nova Scotia B3H 2Y9 Canada

**Keywords:** Sonotubometry, Eustachian tube, Phase-shifting

## Abstract

**Background:**

Sonotubometry is a non-invasive means of assessing Eustachian tube (ET) function. Its interpretation remains a complex task with questionable results due to wide variation between trials. A study was conducted to ascertain whether the measurement of phase shift in sonotubometric signals would be a more reliable indicator of ET patency than fluctuating Sound Pressure Level (SPL).

**Methods:**

The ears of six healthy participants and two participants with patulous ET (PET) were probed with a 100 Hz signal. Five recordings of SPL were performed at the external auditory canal. Cross-correlation was performed among filtered SPL signals and among extracted phase shift waveforms. Peak coefficients were averaged to provide a measure of waveform similarity between trials.

**Results:**

Mean peak cross correlation coefficient for SPL signal measured 0.603 ± 0.057 Standard Error of Mean (SEM) whilst that for Phase-Shift signal measured 0.884 ± 0.027 (SEM). All normal participants demonstrated an observable phase change between the ear and nasal signal during swallowing indicating an acoustic impedance change during the event. For the PET patients tested, the phase measurements in ear and nasal signals follow one another reasonably closely, indicating little or no impedance change during swallowing. It is thought that this impedance change is indicative of opening of the ET in normal patients, and the lack impedance change indicates ET either remaining open or remaining closed throughout the swallow.

**Conclusions:**

Experimental data suggest that phase-shift detection is a more consistent means of interpreting sonotubometric data than SPL analysis.

## Background

The middle ear, a normally sterile chamber, houses the auditory ossicles which couple mechanical vibrations from the external ear to the oval window of the inner ear. Bordered laterally by the tympanic membrane (TM) and medially by the inner ear, ventilation and pressure equalization are achieved through the periodic opening of the Auditory canal or Eustachian tube (ET) [1]. Maximum power transmission occurs when the pressure on either side of the TM are equal – a state described as impedance matched. ET dysfunction may lead to impedance mismatch, and heightened susceptibility to pathological conditions such as TM retraction and subsequent cholesteatoma [2]. Permanently patent ET may cause autophony, tinnitus, and distressing awareness of physiological sounds [3]. Furthermore, permanent patency increases risk of middle ear infection by serving as a route from the nasopharyngeal region through which pathogens may migrate. Children are at a heightened risk as their Eustachian tubes are yet to attain the length, rigidity, and more vertical configuration of adulthood [4].

Presently, auditory tube dysfunction is subjectively assessed, limiting the ability of physicians to detect the early signs of ET dysfunction and objectively assess the efficacy of surgical and medical interventions. Numerous methods have been proposed to objectively visualize/detect the patency of the ET including manometric methods, and conventional imaging techniques [5]. However, one of the more appealing strategies is sonotubometry which relies on the transmission of sound from the nose to the external auditory canal by means of the ET.

The principles of sonotubometry were first described by Politzer in the late 1800’s who reported increased acoustic transmission from a tuning fork through the Eustachian tube during deglutition [6]. Sonotubometry in its modern form arose in the 1950’s when Perlman developed a system to record audio signals and was able to visualize amplitude fluctuations in the recorded signals coinciding with swallowing actions [7]. Swallowing is known to be one of several actions that often, but not always, results in ET opening [1]. Sonotubometry is clinically attractive as a diagnostic tool due to its relative simplicity, wide applicability, safety, and non-invasiveness. Also, the test is performed under physiological conditions. Despite these advantages and decades of development, it has failed to gain traction due to historically poor reproducibility and susceptibility to noise and attenuation. A study by Virtanen revealed significant variation both within and between different sonotubometric techniques [8]. Various strategies have been described in the literature to improve the technique, largely by changing the nature of the stimulus signal: Virtanen [8] explored the efficacy of pure sinusoidal signals at select frequencies; Murti et al. utilized broadband signals [9]; Hori et al. used noise masked signals [10]; and advanced signal encoding strategies have been employed [11]. Sonotubometric data is relatively straightforward to collect, but its interpretation remains a challenge that has caused investigators to question the clinical accuracy/usefulness of the results thus far presented in the data [12].

Many researchers use a chosen (arbitrary) number of standard deviations to classify a recorded section of an SPL signal as either “ET Opening” or “No Opening” [13]. However, these thresholds are not consistent across the literature, and Lindholdt teaches us that SPL changes could be attributable to numerous acoustic phenomena occurring in the nasopharynx during deglutition [12].

Phase-Shift detection for sonotubometric interpretation places less emphasis on changes in the sound pressure level transmitted through the ear and instead focuses on changes in the phase shift of the transmitted signal, caused either by changes in the reactive impedance of the ET or by changes in signal delay, resulting from the collapse or opening of cartilaginous walls of the ET.

In a 1985 patent issued to Meno [14] describing the measurement of ET patency through phase shift detection, Meno reports that the ET has a variable resistance to airflow depending upon the degree of opening during swallowing (deglutition). A consequence of the nasopharynx, middle ear (ME) and the ET being compliant chambers is that their pressure changes are temporally offset. Though coupled in series, the pressure changes in the middle ear will lag pressure changes in the nasal cavity. Consequently, continuous measurement of this lag (or phase difference) could be used to infer the degree of ET opening as a function of time [14]. No experiment was described to test this technique nor were supplemental data provided to assess the efficacy of the technique. The purported advantage in analyzing phase shift was that phase would be less susceptible to distorting factors such as noise, attenuation, and alternate conduction paths.

To lay a framework for sonotubometry that is clinically useful (by being easy to interpret) and reliable (by being less susceptible to interference), this study details a method of analyzing sonotubometric data based on phase detection of the measured signal. The reproducibility and interpretability of this strategy is contrasted against that commonly used in the literature to interpret sonotubometry results.

## Methods

Ethics approval for this study was granted by Capital District Health Authority Research Ethics Board. A 100 Hz sinusoidal signal was generated using LabView (National Instruments, Austin, TX, USA) and fed to a KRK VXT4 Speaker System (Stanton Magnetics, Deerfield Beach, FL, USA) with peak SPL of 107 dB and 45 W power output (frequency response 66 Hz – 22 kHz). The speaker was isolated in a sound proof box and a funnel attached to its diaphragm channeled sound pressure through a ¼” diameter, 1 m long, flexible vinyl tubing to pass sound into an isolated soundbooth. The transmitted signal was captured through a Etymotic Microphone (transducer) placed in the external auditory canal and digitized by a USB-2404 (Measurement Computing Corp., Norton, MA, USA) 24-bit data acquisition system (DAQ) sampling at a rate of 5 kHz and recorded over a 5 s time course. Five separate measurements were performed for each healthy participant. Despite Virtanen’s conclusion that frequencies above 5–6 kHz may be the best to probe for sonotubometry [8], for a sound to be conducted with minimal impedance through a mass, acoustic laws dictate that a narrower tube has lower impedance to low frequencies than to high frequencies [12]. Perlman also reported that a 100 Hz probe tone yielded results more satisfactory than those of higher frequencies [7].

Six participants from the EAR/SENSE lab and two participants from the ET clinic had their measurements taken in the sound booth. The six participants from the EAR/SENSE were otologically healthy and reported no history of chronic ear infection, vestibular dysfunction, nor did they report symptoms of patulous ET or hypofunctioning/insufficient ET. They correspond to Ears 1 to 6. The two participants with PET correspond to Ears 7, and 8. The stimulus was presented to an open nostril and an Etymotic microphone was placed in the contralateral nostril to record the stimulus. Another Etymotic microphone was placed in the ear ipsilateral to the stimulus. Participants were given a glass of water and were signaled to swallow at 1 s following the initiation of recording. For each ear, this process was performed 5 times. The same process was performed on a patient with severe PET confirmed by visualization of TM motility with inspiration.

### Data analysis/signal processing

Experimental data were subsequently processed in MATLAB (Mathworks Inc, Natick, MA, USA). Signal recordings from both the contralateral nostril and ipsilateral ear were passed through a digital 10^th^ order Butterworth Filter with a passband 10 % below and above the frequency of interest (pass band = 90–110 Hz).

The filtered SPL signals were segmented into 10 ms lengths – equal to the period of the 100 Hz stimulus signal. Phase of each 10 ms long segment was calculated and entered as a data point in a new phase array producing 500 datapoints (100/s) over the 5-s long time course. The mean of this array was then subtracted from each data point in the array. Each participant ear yielded 5 SPL signals and 5 phase-shift arrays.

### Method to determine the reliability phase detection method

The five sets of measurements from each patient were compared with one another to yield 10 different cross-correlation products for each of the filtered SPL output and the Phase Shift outputs. Similar waveforms yield higher peak cross-correlation values than dissimilar waveforms. In this manner, the average peak cross-correlation value for a series of waveforms is indicative of the repeatability/stability of the system that produced them. This “system” includes the act of swallowing, nasopharyngeal acoustic changes, and the detection method for the data. Both the amplitude data and the phase data share a common denominator by virtue of being produced by the same set of actions and being captured by the same equipment simultaneously.

## Results

**Fig. 1:**
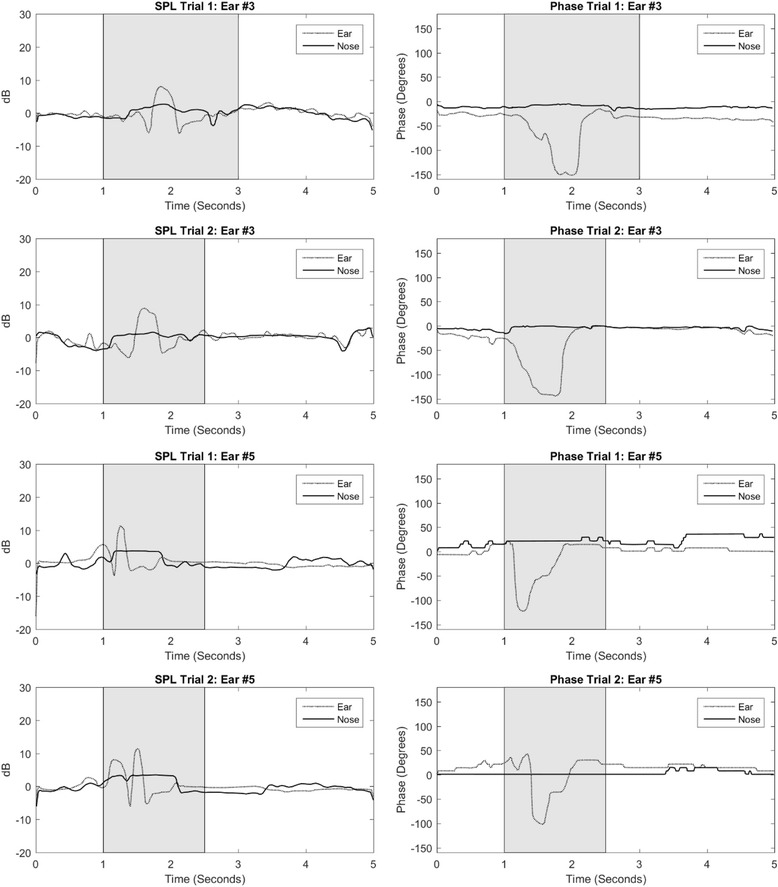
SPL and phase measurements at nose and external auditory canal in selected healthy ears. Participants initiate swallow after a 1-s delay. Shaded region approximates the swallowing duration

Figure 1 shows the sound pressure levels (SPL) and phase shifts measured at both the ears and the noses of selected healthy subjects. Most notable are the phase-shift graphs which trend downward from baseline and return to baseline over the time course coincident with swallowing. The waveforms appear very similar from trial to trial. A large negative phase shift is visualized in the recording at the ear during the swallowing period indicating reduced lag or impedance. This could suggest that there is an opening of the ET during this time period that would coincide with the swallowing action. But one should keep in mind that not every swallowing action opens the Eustachian tube.

**Fig. 2:**
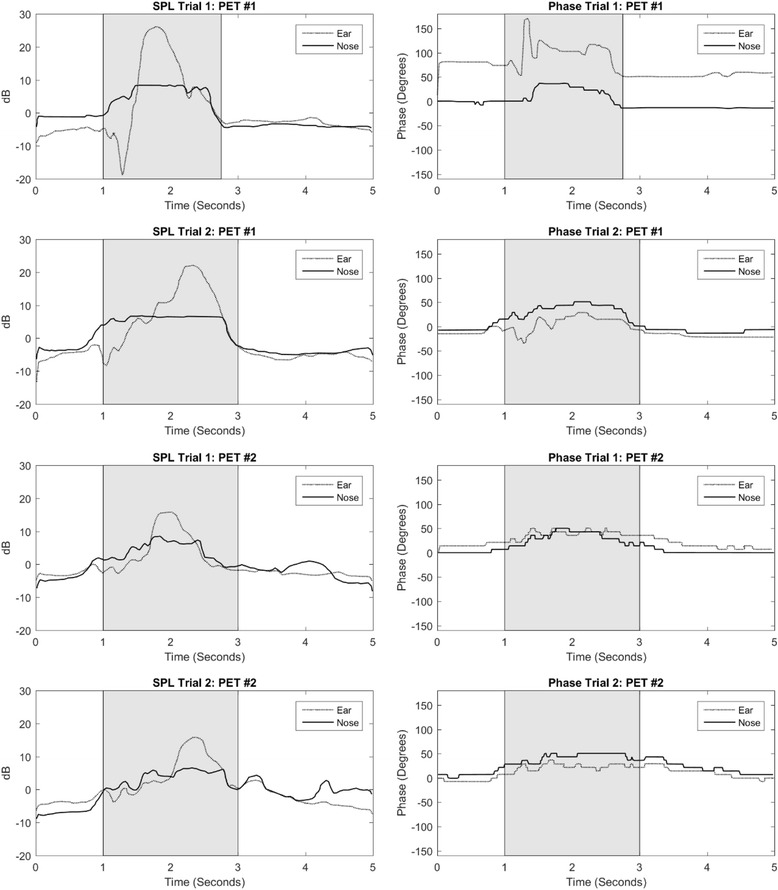
SPL and phase measurements at nose and external auditory canal in two patulous Eustachian tube subjects (confirmed by TM movement on inspiration). Participants initiate swallow after a 1-s delay. Shaded region approximates the swallowing duration

Figure 2 shows the sound pressure levels (SPL) and phase shifts measured at both the ears and the nose of subjects with severe and confirmed cases of PET. PET is verified by visual observation of TM movement with respiration, which indicates direct coupling of the nasopharynx to the ME and thus a patent ET. In this series of images, a high level of similarity between waveforms in the SPL measurements may be observed – less so with the phase measurements. A trend present in the phase measurements of confirmed PET cases, in contrast to those in the healthy subjects in Fig. 1, is that the phase measured at the nose and the external ear canal tend to track one another, i.e. the difference between the two remain unchanged and they therefore move up and down in unison along the 5-s time course. This quasi-synchronized tracking suggests a more direct coupling between the nasopharynx and ME compared to healthy subjects. This finding is congruent with a permanently patent ET.

**Fig. 3:**
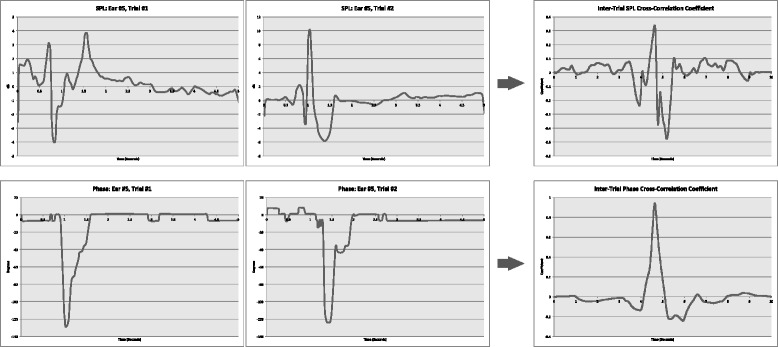
Illustration of the inter-trial cross-correlation coefficient computation

Figure 3 illustrates the process by which the cross-correlation coefficient was calculated between the trials. For each participant ear, the trials were compared with one another to assess the similarity of the graphs. Because there were 5 trials with each participant and a cross correlation is between 2, there are 10 pairings. The more similar the waveforms are, the more narrow and taller the resultant peak and the flatter the baseline. For this particular ear, the peak for the SPL cross-correlation is 0.33 and the peak for the Phase cross-correlation is 0.93.

**Fig. 4:**
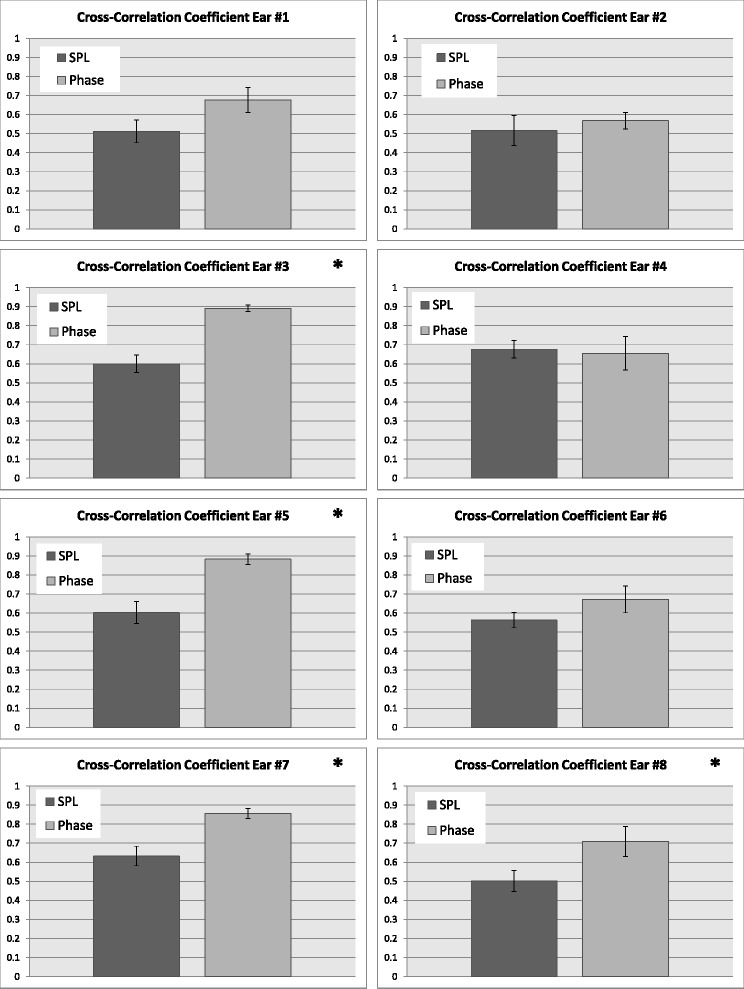
Average peak correlation coefficients with SPL and phase measurements. Subfigures attaining statistical significance (*p* < 0.05) are denoted with an asterisk

For each participant ear, the maximum values from each cross-correlation trial were averaged. Across all participants, mean peak cross correlation coefficient for SPL signal measured 0.603 ± 0.057 (SEM) whilst that for Phase-Shift signal measured 0.884 ± 0.027 (SEM). Figure 4 shows bar graphs of average peak cross-correlation value for phase vs. amplitude (error bars are SEM). In all datasets attaining statistical significance (*p* < 0.05, unpaired t-test), the peak cross-correlation coefficient in phase is greater than that in SPL.

These patients with PET illustrate that an increase or peak in SPL can still be achieved with swallowing but there is no sudden phase separation. We do not see the characteristic phase dip seen on the graphs from healthy patients.

Overall, the results of this experiment show that the difficulty of interpreting sonotubometry data is compounded by the variability in SPL levels and highlights the comparative relative stability of phase data.

## Discussion

Sonotubometry is an attractive clinical tool, yet remains widely unused in such settings. Previous attempts have used the detection of SPL and their standard deviation from the mean to infer the state of the ET, however the SPL of the test tone may or may not be consistent in the nasopharynx thus complicating the interpretation of the SPL data at the external auditory canal. Sinusoidal signals have been the predominant signal used to probe the ET and although the phase of these signals may be influenced by a number of factors, our early results suggest that the influence and the resultant measurements are more consistent than signal amplitude or SPL.

The work presented in this paper models the nasopharyngeal region, ET and middle ear as compliant chambers in series and highlights the possible utility of using dynamic/shifting phase differences to determine the state of the ET and the degree to which it is open. Using a low frequency (100 Hz) probing signal and a DAQ to capture SPL at participants’ ears and noses, notable deviations were observed when the participants were instructed to swallow. In the majority of otologically healthy patients, a negative phase shift occurred during the swallowing motion. The phase detected at the ear deviated sharply from the phase measured in the nose which itself remained relatively flat. This shift may be indicative of decreased impedance experienced by the transmitted acoustic signal (presumably through opening of the ET). However, it could also be due in part to stiffening of the tissue sound bridge (e.g. the articulation of the Temporomandibular Joint), the movement of the soft palate, the movement of the tongue, muscles of the larynx, the temporarily increased pressure in the nasopharyngeal cavity, etc. The nature and magnitude of the contribution of the ET to this phase shift is yet to be determined and is the subject of further research.

The phase shifts measured at the ears and noses of the subjects with confirmed PET differ from those of healthy subjects in that the nasal phase also deviated from the baseline during the act of swallowing. Due to the complex changes that occur in the nasopharyngeal space during swallowing, it is difficult to interpret the significance of this deviation. More meaningful, however, is the fact that the ear and nasal phases tracked together during the act of swallowing – that is, they trended upward and downward in relative unison/synchrony. This synchrony, and upward deflection corroborate the model of directly coupled chambers in PET and could perhaps be one of the discriminating factors to differentiate between PET patients and healthy subjects.

## Conclusions

Despite the small sample size used in this preliminary study, the results, indicating improved consistency using phase data relative to SPL in sonotubometry, are compelling and warrant further investigation. To the best of the authors’ knowledge, at the time of this preparation there had been no studies detailing phase-shift detection as a strategy for sonotubometry data analysis and presenting experimental results. Yet to be determined are the method’s sensitivity and specificity for transient ET opening and PET which may be accomplished using a detection protocol described by Swarts et al. [15]. With demonstrable repeatability, interpretability, and sufficient specificity and sensitivity, sonotubometry will maintain the potential to become a widely adopted clinical tool.

## Abbreviations

ET: eustachian tube
DAQ: data acquisition device
PET: patulous eustachian tube
SEM: standard error of mean
SPL: sound pressure level
TM: tympanic membrane
